# Modified Dynamic Decode-and-Forward Relaying Protocol for Type II Relay in LTE-Advanced and Beyond

**DOI:** 10.1371/journal.pone.0167457

**Published:** 2016-11-29

**Authors:** Sung Sik Nam, Mohamed-Slim Alouini, Seyeong Choi

**Affiliations:** 1 Department of Electronics Engineering, Hanyang University, Seoul, Korea; 2 Department of Electrical Engineering, KAUST, Thuwal, Makkah Province, Saudi Arabia; 3 Department of Information and Communication Engineering, Wonkwang University, Jeonbuk, Korea; West Virginia University, UNITED STATES

## Abstract

In this paper, we propose a modified dynamic decode-and-forward (MoDDF) relaying protocol to meet the critical requirements for user equipment (UE) relays in next-generation cellular systems (e.g., LTE-Advanced and beyond). The proposed MoDDF realizes the fast jump-in relaying and the sequential decoding with an application of random codeset to encoding and re-encoding process at the source and the multiple UE relays, respectively. A subframe-by-subframe decoding based on the accumulated (or buffered) messages is employed to achieve energy, information, or mixed combining. Finally, possible early termination of decoding at the end user can lead to the higher spectral efficiency and more energy saving by reducing the frequency of redundant subframe transmission and decoding. These attractive features eliminate the need of directly exchanging control messages between multiple UE relays and the end user, which is an important prerequisite for the practical UE relay deployment.

## Introduction

Relay transmission can help increase both the cell coverage and the data rate of the cutting edge cellular systems without creating undue inter-cell interference, and as such, has been considered in the latest cellular standards (e.g., LTE-Advanced) [1]. Mainly, two types of relaying strategies, namely type I or infrastructure relay [2–4] and type II or user equipment (UE) relay [2, 5–9] have been investigated. Recent trend shows that rigorous studies have been done in various relay models which cover the channel/resource management to meet QoSs [10–17].

**Fig 1:**
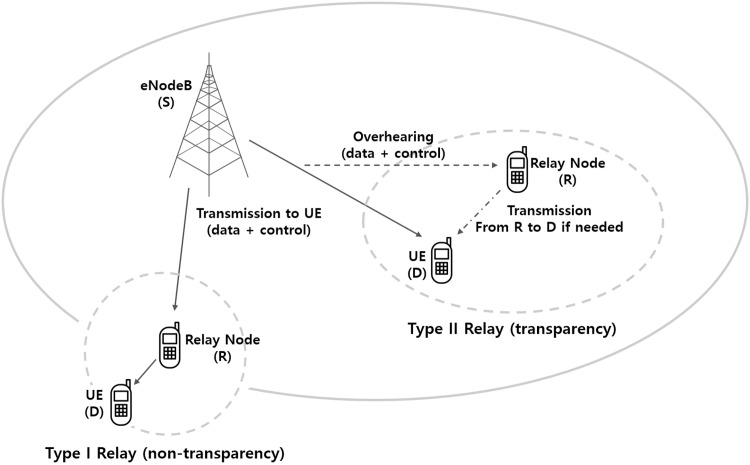
Example of Type I and Type II relays.

In the 3rd generation partnership project (3GPP) [1], the type I relay represents two hop half-duplex relaying (or non-transparent relay) scheme while the type II relay describes multicast cooperative relaying (or transparent relay) scheme. As shown in Fig 1, the type I relay essentially creates an independent cell with a small coverage resulting in the coverage extension, whereas the type II relay increases the user data rate by forwarding overheard messages. The current LTE-Advanced specification does not define any detailed functionality of the type II relay. This is because it was decided to focus on the type I relay during the standardization of Release 10 and table the type II relay as a study item for the future releases in 3GPP. However, [1] summarizes baseline requirements about the type II relay such as “the relay-to-destination (R-D) link must be operated in an open-loop (or transparent) mode because of no dedicated control channel”. More specifically, the R-D link channel state information (CSI) is not available at UE relays and each UE relay node appears transparent to the end user. Consequently the end user can not distinguish among signals transmitted from the source and the relays. Therefore, under the open-loop transmission assumption, how to relay the received data in UE relays to the destination is a still challenging problem.

It is commonly known in the literature that there are two major conventional relaying protocols, namely amplify-and-forward (AF) and decode-and-forward (DF) protocols [2]. The main drawback of the AF protocol is the amplification of the unwanted signal (e.g., noise and interference) at the relay. Meanwhile, the DF protocol can introduce an error propagation from R to D. To overcome this drawback, Azarian *et al*. [5] proposed the dynamic DF (DDF) protocol where the relay switches to a transmission mode only after it has correctly decoded the message broadcasted by the source. One of the main advantages of the DDF protocol is that the fast jump-in relaying and the joint decoding schemes are available at the relays and the end user, respectively. However, the joint decoding at the end user requires the knowledge of relay forwarding times while the forwarding time at the relay is random because of the random nature of the source-to-relay (S-R) link quality. Therefore, the conventional DDF protocol [5] is not compatible to the next generation cellular systems, especially type II relay based systems.

Following these observations, we propose a modified DDF (MoDDF) protocol for the type II relay. Our proposed MoDDF is suitable for UE relays where no control message could be exchanged directly between UE relay and the end user. With MoDDF, the end user adopts joint and sequential decoding as well as early termination of decoding to significantly save the resource. To realize the fast jump-in relaying and decoding, a subframe based sequential relaying and decoding is employed. Further, to enable joint decoding at the end user with the open-loop retransmissions from multiple UE relays, we propose several subchannel/code selection strategies based on the rateless codes at UE relays [18].

The main results of the paper are summarized as follows:

- With the proposed relaying protocol, each relay and the end user can attempt to perform the subframe-by-subframe message decoding. In addition, the proposed scheme eliminates the need of directly exchanging control messages between multiple UE relays and the end user, which is an important prerequisite for the practical UE relay deployment. The end user can blindly search for the forwarded messages from UE relays based on the pre-determined subchannel/code selection strategies. After receiving each subframe, the destination node performs the joint and sequential subframe-based decoding.
- As an additional benefit, the data rate at the end user can be increased through the energy, information, and mixed combining (EC, IC, and MC) in each subframe [19, 20]. The information combining across subframes results from the subframe based joint and sequential decoding. As a result, the possibility of successful decoding at the end user before receiving a whole frame is likely to improve.
- The spectrum efficiency can be improved by adopting the early termination protocol based on the overhearing mechanism. With our proposed scheme, when the end user succeeds in decoding, the end user shall send ACK to S and typically, UE relays can overhear ACK. Both S and UE relays that participate in the retransmission shall terminate their on-going transmissions. Such a possible early termination of decoding at the end user will help improve the spectrum efficiency by reducing the redundant subframe transmission. Additionally, with the help of the overhearing mechanism, the spectrum efficiency can be further improved by limiting the forwarding UE relays to a subset of candidate relays based on the quality of the R-D link.

The rest of this paper is organized as follows. Section II describes the proposed protocol, including the mode of operation, the subchannel/code selection strategies, and the overhearing mechanism. Section III addresses the performance analysis of the proposed MoDDF relaying protocol. Specifically, we focus on the achievable rate of the two proposed subchannel/code selection strategies. Further, to improve the spectral efficiency of the proposed MoDDF protocol, the overhearing mechanism based relaying protocol and related performance analyses are considered in Section IV. Finally, Section V illustrates numerical results via some selected figures and Section VI provides some concluding remarks.

## Proposed MoDDF Relaying Protocol

### System Model

We consider a relay-based wireless network consisting of a source (S), a destination (D) and multiple UE relays (Rs), each of which has a single antenna. We assume that half-duplex UE relays can overhear the reference signals exchanged between S and D. The transmission process is organized in two phases. In the first phase (listening phase), S broadcasts its message, and D and Rs receive it. If at least one R successfully decodes before D, the second phase (collaboration phase) starts, in which both S and R transmit the message to D [20].

**Fig 2:**
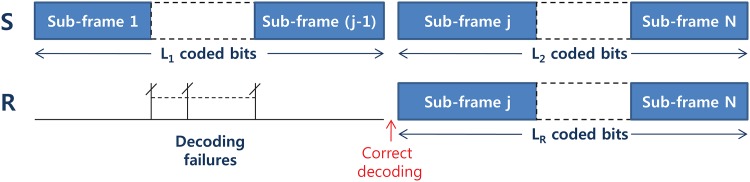
Frame structure.

Specifically, the information bits are encoded by a rateless code at S to form a frame [20]. The frame is then segmented into a number of concatenated subframes of same length, as shown in Fig 2 and transmitted sequentially. The UE relays and D will attempt to decode the information after receiving each subframe. Since the S-R links are statistically independent, we expect that correct decoding of the received message at UE relays can occur randomly at an arbitrary subframe index. Fig 2 illustrates the case that a frame is segmented into *N* subframes, and correct decoding at the *i*-th UE relay occurs after receiving (*j* − 1) subframes for *j* < *N* [21]. Note that the length of *L*_1_ and *L*_2_ can be varied. It is noteworthy that the subframe index 1, 2, ⋯, *N* in Fig 2 are not necessarily contiguous in time domain and each transmission from S or R to D can be scheduled in an arbitrary manner, possibly accompanied by a control data which delivers the detailed scheduling information to D.

We assume that orthogonal resources are available for S-D and R-D channels. In this paper, code division multiple access (CDMA) signaling scheme is adopted for the orthogonal resource allocation to S and Rs [20]. Finally, it is assumed that all channels (both S-D and S-R-D) experience quasi-static independent and identically distributed (i.i.d.) Rayleigh fading. The fading coefficients remain constant at least during the transmission of a frame and are independent from one frame to another.

### Mode of Operation of MoDDF Relaying Protocol

Suppose that correct decoding may be achieved after receiving (*j* − 1) subframes where *j* ≤ *N*. For practical implementation with the open-loop retransmission constraint over multiple R-D links, we design the transmission strategies based on the rateless codes as follows:

1. S employs a pre-determined sequence of subchannels (i.e., spreading codes/sequences for CDMA) and codes (i.e., generating vectors for the rateless code) to transmit the subframes. We assume that these subchannel code sequences information are a priori known to UE relays and D.
2. S transmits the encoded messages to D while each UE relay overhears them. When the correct message decoding occurs at the *i*-th UE relay after receiving (*j* − 1) subframes, this relay begins to re-encode and then forward the message to D at the *j*-th subframe duration using the pre-determined subchannel/codes sequences.
3. Since the forwarding times of UE relays are unknown to D, D blindly searches for the forwarded message based on the pre-determined subchannel sequence. Then, D combines the received subframes from S and UE relays to decode the transmitted packet.
4. As soon as D succeeds in decoding, D may inform S with an ACK while UE relays can overhear it. Then both S and UE relays (involved in the retransmission) terminate their transmission.

### Subchannel and Code Selection Strategies for Relay Terminal

**Fig 3:**
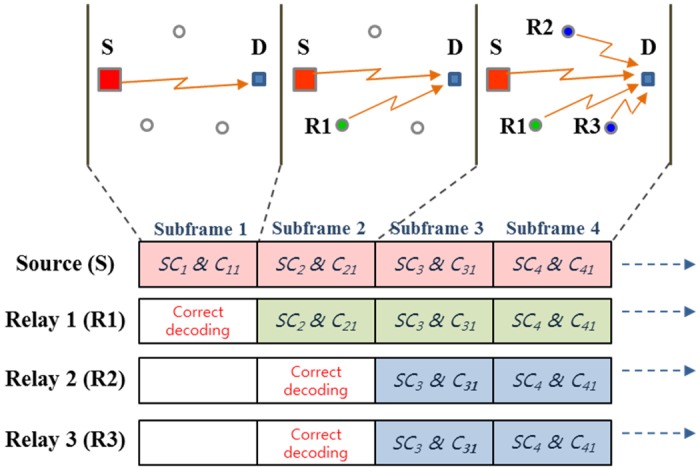
In-phase selection strategy (MoDDF_IPS_).

**Fig 4:**
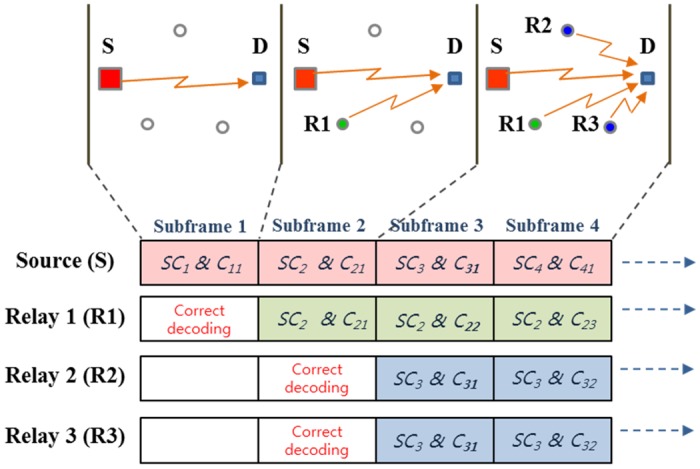
Fixed random selection strategy (MoDDF_FS_).

Figs 3 and 4 illustrate examples of each proposed subchannel/code selection strategies considered in this work, especially considering the CDMA-based system. For the better explanation, we adopted a simplified model such as three participating relays with four subframes. Here, *SC*_*i*_ represents the *i*-th subchannel and *C*_*k*,*l*_ represents the *l*-th subframe of the generated data (*l* ≤ *N*) using the *k*-th rateless code generating vector. Different generating vectors are applied across subframes and the active UE relay always starts transmitting the first portion of the encoded data. The two proposed strategies are defined as follows:

- **In-phase Strategy (MoDDF_IPS_):** R, upon successful decoding the (*j* − 1)-th subframe, starts retransmitting the first subframe on the same subchannel used by S using the same generating vectors (e.g., *C*_*j*,1_, *C*_*j*+1,1_, ⋯) as illustrated in Fig 3.
- **Fixed Strategy (MoDDF_FS_):** R, upon successful decoding the (*j* − 1)-th subframe, starts retransmitting, on the subchannel used by S at the *j*-th subframe (e.g., *j*-th subchannel), the coded subframe with the *j*-th generate vector (e.g., *C*_*j*,1_, *C*_*j*,2_, ⋯) as illustrated in Fig 4.

We assume that the same transmission attributes as S (e.g., modulation scheme, coding scheme, reference signal type, scrambling sequence, etc.) is applied to the signal forwarded by the relay. S may inform these attributes to UE relays in advance. With the above transmission strategies, energy combining (EC) is possible when both S and UE relays are applying the same subchannel/code in a given subframe. Because S-D and R-D channels have different propagation delays, D can apply a Rake receiver for (maximal-ratio) combining signals from S and UE relays. Information combining (IC) is implemented when relays with distinct forwarding times use different subchannels/codes. In this case, due to the different spreading codes, D can distinguish signals from S and UE relays and then the S-D and R-D links are information combined as they use the different generating vectors [22]. Otherwise, mixed combining (MC) is performed at D, where signals from S and some of relay(s) with the same subchannel/code are energy combined while signals transmitted from some of relays with different subchannels/codes are information combined. For example of MoDDF_IPS_ in Fig 3, R1, upon successful decoding the first subframe, starts the retransmission in the second subframe with the same subchannel/code used by S which leads to EC. Similarly, R2 and R3, upon successful decoding the second subframe, start the retransmission in the third subframe with the same subchannel/code used by S. In case of MoDDF_FS_ in Fig 4, R1, upon successful decoding the first subframe, starts the retransmission in the second subframe with the same subchannel/code used by S which leads EC. Then, R2 and R3, upon successful decoding the second subframe, start the retransmission in the third subframe. In this case, S, R2, and R3 use the same subchannel/code but R1 uses the different subchannel/code in the third subframe. As a result, MC is performed. Note that for both examples, IC is performed across subframes without combining any repeated information.

## Average Achievable Rate

In this section, we focus on the analysis of the achievable rate (*AR*). For the analytical tractability, the possible early termination is not considered for now but its performance improvement capability will be shown later with the simulation results.

According to the mode of operation of the proposed scheme, the execution of direct transmission (DT), EC, IC and MC, and their combination depends entirely on the number of active relays at current and all previous subframes. The instantaneous rate over the *j*-th subframe for each case can be obtained as follows:

- DT occurs when the number of activated relays at all previous subframes and current subframe is 0, providing the instantaneous rate as log(1 + *γ*_SD_) where *γ*_SD_ is the signal-to-noise ratio (SNR) of the S-D link.
- EC occurs when the number of activated relays at all previous subframes is 0 but not 0 at current subframe, providing the instantaneous rate as $\log(1+\sum_{i}^{R_{j}}\gamma_{i})$ where *γ*_*i*_ is the instantaneous SNR received at D and *R*_*j*_ is the number of new active relays at the *j*-th subframe (*j* = 1, 2, ⋯, *N*) which only depends on the S-R link conditions.
- IC occurs when relays are activated at one or more previous subframes and there is only one activated relay at each of those subframes, but 0 at current subframe. Hence, the instantaneous rate is $\sum_{i}^{R_{j}}\log(1+\gamma_{i})$.
- MC occurs otherwise, providing the instantaneous rate as $\sum_{l}^{j}\log(1+\sum_{i}^{R_{l}}\gamma_{i})$.

As such, if we let $AR(\bar{\gamma }|R_{1}=n_{1},\cdots ,R_{N}=n_{N})$ be the conditional average AR per frame at a given *R*_*j*_ as a function of $\bar{\gamma }$ which is the common average SNR of the received signal at D, an average *AR*, $\bar{AR}$, per frame can be derived based on the given number of new active relays at each subframe, *R*_*j*_, as

$$\begin{array}{c} \bar{AR}=\sum_{n_{1}=0}^{M}\sum_{n_{2}=0}^{M-n_{1}}\cdots \sum_{n_{N}=0}^{M-(n_{1}+\cdots +n_{N_{}-1})}AR(\bar{\gamma }|R_{1}=n_{1},\cdots ,R_{N}=n_{N})\;p_{R_{1},\cdots ,R_{N}}\left(n_{1},\cdots ,n_{N}\right) \end{array}$$ (1)

where *M* is a total number of active relays, *p*_*R*_1_, ⋯, *R*_*N*__(*n*_1_, ⋯, *n*_*N*_) is the joint probability mass function (PMF) of *R*_*j*_, and $\bar{\gamma }$ is a common average SNR of the received signal at D. In order to simplify the analysis, we assume the Rs-D and S-D links are identical. However, in practice the Rs-D and S-D links are not identical, which can be reflected into our result in Eq (10). Note that if the channel conditions (especially the S-Rs link conditions) are the same, then the number of new active relays of both MoDDF_IPS_ and MoDDF_FS_ at each subframe is the same.

### Joint PMF of *R*_*j*_

Since the distribution of *R*_*j*_ is only affected by the sum of the new active relays in the previous subframes, the joint PMF of *R*_*j*_ can be obtained as the product of the PMF of *R*_1_, the conditional PMF of *R*_2_ given *R*_1_, the conditional PMF of *R*_3_ given *R*_1_ and *R*_2_, and so on. As a result, the target joint PMF of *R*_*j*_ in Eq (1) can be written as

$$\begin{array}{ccc} & & p_{R_{1},\cdots ,R_{N}}\left(n_{1},\cdots ,n_{N}\right)\\ & & \;=p_{R_{1}}\left(n_{1}\right)p_{R_{2}|R_{1}=n_{1}}\left(n_{2}\right)p_{R_{3}|R_{2}=n_{2},R_{1}=n_{1}}\left(n_{3}\right)\cdots p_{R_{N}|R_{N-1}=n_{N-1},\cdots ,R_{1}=n_{1}}\left(n_{N}\right). \end{array}$$ (2)

Note that during the first subframe (*j* = 1), only S transmits a signal to D which means there are no activated relays, i.e., only DT works, *R*_1_ = 0. Therefore, we can rewrite Eq (2) as

$$\begin{array}{ccc} & & p_{R_{1},\cdots ,R_{N}}\left(n_{1},\cdots ,n_{N}\right)\\ & & \;=p_{R_{2}}\left(n_{2}\right)p_{R_{3}|R_{2}=n_{2},}\left(n_{3}\right)\cdots p_{R_{N}|R_{N-1}=n_{N-1},\cdots ,R_{2}=n_{2}}\left(n_{N}\right) \end{array}$$ (3)

where *R*_2_ follows the discrete probability distribution of the number of relays that succeeded in decoding, i.e., a binomial distribution, as

Pr(R2=n2)=(Mn2)p2n2(1-p2)M-n2. (4)

Here, *p*_2_ is the probability that the relay is newly activated at the second subframe (i.e., The relay, upon successful decoding the first subframe, starts retransmitting from the second subframe.). Therefore, if we denote *γ*_SR_ as the SNR of the S-R link whose probability density function (PDF) is *f*_*γ*_SR__(*γ*) and *R*_th_ as the rate threshold for decoding at the receiver, we can obtain

$$\begin{array}{ccc} p_{j} & = & \mathrm{Pr}[R_{\text{th}}\leq \left(j-1\right)\times \log\left(1+\gamma_{\text{SR}}\right)]\\ & = & \mathrm{Pr}[\gamma_{\text{SR}}\geq \exp\left(\frac{R_{\text{th}}}{j-1}\right)-1]\\ & = & \int_{\exp(\frac{R_{\text{th}}}{j-1})-1}^{\infty }f_{\gamma_{\text{SR}}}\left(\gamma \right)d\gamma . \end{array}$$ (5)

The conditional PMF of *R*_3_ given *R*_2_ can be written as

$$\begin{array}{c} \mathrm{Pr}\left(R_{3}=n_{3}|R_{2}=n_{2}\right)=\frac{\mathrm{Pr}(R_{3}=n_{3},R_{2}=n_{2})}{\mathrm{Pr}(R_{2}=n_{2})} \end{array}$$ (6)

which is given in the following closed-form as

Pr(R3=n3|R2=n2)=(M-n2n3)(p3′1-p2)n3(1-p3′1-p2)M-n2-n3 (7)

where

$$\begin{array}{ccc} p_{j^{\prime }} & = & \mathrm{Pr}[\left(j-2\right)\times \log\left(1+\gamma_{\text{SR}}\right)<R_{\text{th}}\leq \left(j-1\right)\times \log\left(1+\gamma_{\text{SR}}\right)]\\ & = & \mathrm{Pr}[\gamma_{\text{SR}}\geq \exp\left(\frac{R_{\text{th}}}{j-1}\right)-1\;\text{and}\;\gamma_{\text{SR}}<\exp\left(\frac{R_{\text{th}}}{j-2}\right)-1]\\ & = & \int_{\exp(\frac{R_{\text{th}}}{j-1})-1}^{\exp(\frac{R_{\text{th}}}{j-2})-1}f_{\gamma_{\text{SR}}}\left(\gamma \right)d\gamma . \end{array}$$ (8)

Note that the conditional PMF of *R*_*j*_ depends only on the given total number of active relays at the (*j* − 1)-th subframe. Therefore, generalizing the above special cases, we can obtain the conditional PMF for a general case (*j* > 2) as

Pr(Rj=nj|Rj-1=nj-1,⋯,R2=n2)=Pr(Rj=nj,Rj-1=nj-1,⋯,R2=n2)Pr(Rj-1=nj-1,⋯,R2=n2)=Pr(Rj=nj,Rj-1=nj-1,⋯,R2=n2)Pr(Rj-1+⋯+R2=nj-1+⋯+n2)=(M-n2-⋯-nj-1nj)(pj′1-pj-1)nj(1-pj′1-pj-1)M-n2-⋯-nj. (9)

### Conditional Average Achievable Rate

The conditional average *AR* in Eq (1), $AR(\bar{\gamma }|\cdot )$, can be derived for the given number of new active relays, *R*_*j*_, at the *j*-th subframe (*j* = 1, 2, ⋯, *N*) as

$$\begin{array}{c} AR(\bar{\gamma }|R_{1}=n_{1},\cdots ,R_{N}=n_{N})=\sum_{k=1}^{N}\int_{0}^{\infty }\lambda_{k}f_{\lambda_{k}}\left(\lambda_{k}\right)\;d\lambda_{k} \end{array}$$ (10)

where *λ*_*k*_ and *f*_*λ*_*k*__(*λ*_*k*_) are an instantaneous *AR* and its PDF conditioned on the number of active relays at the *k*-th subframe. For the analytical convenience, two cases, (*k* = 1) and (*k* > 1), are considered separately. For a given total number of active relays, Eq (10) can be re-written as

$$\begin{array}{c} \sum_{k=1}^{N}\int \lambda_{k}f_{\lambda_{k}}\left(\lambda_{k}\right)\;d\lambda_{k}=\int \lambda_{1}f_{\lambda_{1}}\left(\lambda_{1}\right)\;d\lambda_{1}+\sum_{k=2}^{N}\int \lambda_{k}f_{\lambda_{k}}\left(\lambda_{k}\right)\;d\lambda_{k}. \end{array}$$ (11)

In what follows, we derive *λ*_*k*_ and *f*_*λ*_*k*__(*λ*_*k*_) for each strategy.

#### MoDDF_IPS_

1. For *k* = 1:
  In this case, only DT is performed. Thus, the instantaneous *AR* at the first subframe, *λ*_1_, can be written as
  $$\begin{array}{c} \lambda_{1}=\log\left(1+\gamma_{\text{SD}}\right). \end{array}$$ (12)
  Let *λ*_1_ = log(1 + *γ*), then $\gamma =e^{\lambda_{1}}-1\;\text{for}\;\text{nats}\;\left(2^{\lambda_{1}}-1\;\text{for}\;\text{bits}\right)$ and the derivative is $\frac{d\gamma }{d\lambda_{1}}=e^{\lambda_{1}}\;\text{for}\;\text{nats}\;\left(2^{\lambda_{1}}\ln2\;\text{for}\;\text{bits}\right)$. As a result, *f*_*λ*_1__(*λ*_1_) can be written as
  $$\begin{array}{c} f_{\lambda_{1}}\left(\lambda_{1}\right)=f_{\gamma }\left(e^{\lambda_{1}}-1\right)\cdot e^{\lambda_{1}}. \end{array}$$ (13)
  Note that for i.i.d. Rayleigh fading conditions, *γ* follows an exponential distribution [23]. Therefore, the PDF of the instantaneous *AR* conditioned on the number of active relays at the first subframe, *f*_*λ*_1__(*λ*_1_), is given by
  $$\begin{array}{c} f_{\lambda_{1}}\left(\lambda_{1}\right)=\frac{1}{\bar{\gamma }}\exp\left(-\frac{\exp\left(\lambda_{1}\right)-\bar{\gamma }\lambda_{1}-1}{\bar{\gamma }}\right). \end{array}$$ (14)
2. For *k* > 1:
  In this case, EC may occur at D. Thus, *λ*_*k*_ for *k* > 1 at D can be written as
  $$\begin{array}{c} \lambda_{k}=\log\left(1+{\gamma^{\prime }}_{k}\right) \end{array}$$ (15)
  where
  $$\begin{array}{c} {\gamma^{\prime }}_{k}=\sum_{j=1}^{k}\left(\sum_{i=1}^{R_{j}}\gamma_{j,i}\right)+\gamma_{\text{SD}}. \end{array}$$ (16)
  Similarly, *f*_*λ*_*k*__(*λ*_*k*_) can be written as
  $$\begin{array}{c} f_{\lambda_{k}}\left(\lambda_{k}\right)=f_{{\gamma^{\prime }}_{k}}\left(e^{\lambda_{k}}-1\right)\cdot e^{\lambda_{k}}\;\text{for}\;\;{\gamma^{\prime }}_{k}\neq 0. \end{array}$$ (17)
  For i.i.d. Rayleigh fading conditions, *γ*_*j*,*i*_ and *γ*_SD_ are identical exponential random variables (RVs) with parameter $\frac{1}{\bar{\gamma }}$. Then, $\sum_{i=1}^{R_{j}}\gamma_{j,i}$ is a gamma RV with parameters $\left(R_{j},\frac{1}{\bar{\gamma }}\right)$. Meanwhile, if *X*_1_, *X*_2_, ⋯, *X*_*n*_ are independent gamma RVs with parameters (*t*_*i*_, *λ*), then the sum of these RVs follows a Gamma distribution with parameters $\left(\sum_{i=1}^{n}t_{i},\lambda \right)$. As results, *γ*′_*k*_ follows a Gamma distribution with parameters $\left(N_{k},\frac{1}{\bar{\gamma }}\right)$, where $N_{k}=\sum_{j=1}^{k}R_{j}+1$. Therefore, *f*_*λ*_*k*__(*λ*_*k*_) can be specialized to
  fλk(λk)=1Γ(Nk)γ¯Nk(exp(λk)-1)Nk-1exp(-exp(λk)-1γ¯)exp(λk)=∑l=0Nk-1(Nk-1l)(-1)Nk-1-lΓ(Nk)γ¯Nkexp((l+1)λk)exp(-exp(λk)-1γ¯) (18)
  where Γ(⋅) is a Gamma function [24, Eq (8.310.1)].

#### MoDDF_FS_

1. For *k* = 1:
  In this case, *λ*_1_ and *f*_*λ*_1__(*λ*_1_) have the same results as MoDDF_IPS_.
2. For *k* > 1:
  In this case, *λ*_*k*_ depends on the given number of active relays at both all previous (*j*-th for 1 ≤ *j* < *k*) and current (*k*-th) subframes. In the latter case, an additional S-D link should be considered. As results, *λ*_*k*_ can be written as
  $$\begin{array}{c} \lambda_{k}=\sum_{j=1}^{k-1}\lambda_{k,j}+\lambda_{k,k} \end{array}$$ (19)
  where
  $$\begin{array}{c} \lambda_{k,j}=\log\left(1+{\gamma^{\prime }}_{j}\right) \end{array}$$ (20)
  and
  $$\begin{array}{c} {\gamma^{\prime }}_{j}=\left\{ \begin{array}{cc} \sum_{i=1}^{R_{j}}\gamma_{j,i} & \text{for}\;\;1\leq j<k\\ \sum_{i=1}^{R_{k}}\gamma_{k,i}+\gamma_{\text{SD}} & \text{for}\;\;j=k. \end{array}\right. \end{array}$$ (21)
  Note that *f*_*λ*_*k*__(*λ*_*k*_) can be derived with the help of the Jacobian transformation and the characteristic function (CF) [23]. Here, *λ*_*k*,*j*_ are independent to each other. Therefore, if we let the CFs of *λ*_*k*_ and *f*_*λ*_*k*,*j*__(*λ*_*k*,*j*_) be *M*_*λ*_*k*__(*jω*) and *M*_*λ*_*k*,*j*__(*jω*), respectively, then the CF of *λ*_*k*_ (*λ*_*k*_ = *λ*_*k*,1_ + *λ*_*k*,2_ + ⋯ + *λ*_*k*,*k*_) can be obtained as
  $$\begin{array}{c} M_{\lambda_{k}}\left(j\omega \right)=M_{\lambda_{k,1}}\left(j\omega \right)M_{\lambda_{k,2}}\left(j\omega \right)\cdots M_{\lambda_{k,k}}\left(j\omega \right) \end{array}$$ (22)
  where $M_{\lambda_{k,j}}\left(j\omega \right)=\int_{0}^{\infty }f_{\lambda_{k,j}}\left(x\right)\exp\left(j\omega x\right)dx$. With Eq (22), we derive the PDF of *λ*_*k*,*j*_ which is given as
  $$\begin{array}{c} f_{\lambda_{k,j}}\left(\lambda_{k,j}\right)=f_{{\gamma^{\prime }}_{j}}\left(e^{\lambda_{k,j}}-1\right)\cdot e^{\lambda_{k,j}}\;\text{for}\;\;{\gamma^{\prime }}_{j}\neq 0. \end{array}$$ (23)
  Here, similar to the previous cases, especially for 1 ≤ *j* < *k*, *γ*′_*j*_ follows a Gamma distribution with parameters $\left(R_{j},\frac{1}{\bar{\gamma }}\right)$ [23] over i.i.d. Rayleigh fading assumptions. Hence, we can express Eq (23) as
  fλk,j(λk,j)=∑l=0Rj-1(Rj-1l)(-1)Rj-1-lΓ(Rj)γ¯Rj×exp((l+1)λk,j)exp(-exp(λk,j)-1γ¯). (24)
  To obtain the CF of *λ*_*k*,*j*_, we first evaluate the following integration of a double exponential formula.
  $$\begin{array}{c} \int_{0}^{\infty }\exp(-\frac{1}{\bar{\gamma }}\left(\exp\left(x\right)-\bar{\gamma }\left(s+l+1\right)x-1\right))dx. \end{array}$$ (25)
  Let exp(*x*) = *t*, then *x* = ln *t* and $dx=\frac{1}{t}dt$. Therefore, Eq (25) can be re-written as
  $$\begin{array}{c} \int_{1}^{\infty }\exp\left(-\frac{t}{\bar{\gamma }}\right)t^{s+l}\exp\left(\frac{1}{\bar{\gamma }}\right)dt=\exp\left(\frac{1}{\bar{\gamma }}\right)\int_{1}^{\infty }t^{s+l}\exp\left(-\frac{t}{\bar{\gamma }}\right)dt. \end{array}$$ (26)
  Then, based on the generalized exponential integral equation [25, 5.1.4], the closed-form expression of Eq (25) can be obtained as
  $$\begin{array}{c} \exp\left(\frac{1}{\bar{\gamma }}\right)\int_{1}^{\infty }t^{s+l}\exp\left(-\frac{t}{\bar{\gamma }}\right)dt=\exp\left(\frac{1}{\bar{\gamma }}\right){\text{E}}_{-s-l}\left(\frac{1}{\bar{\gamma }}\right) \end{array}$$ (27)
  where E_*n*_(*x*) is the exponential integral function, E_*n*_(*x*) = *x*^*n*−1^Γ(1 − *n*, *x*). With the help of Eq (27), the CF expression, *M*_*λ*_*k*,*j*__(*jω*), can be obtained as
  Mλk,j(s)=∫0∞fλk,j(x)exp(sx)dx,=∑l=0Rj-1(Rj-1l)(-1)Rj-1-lΓ(Rj)γ¯Rjexp(1γ¯)E-l-s(1γ¯). (28)
  Inserting Eqs (28) to (22), we can obtain the closed-form expression of the multiple product of CFs as
  Mλk(s)=∏j=1kMλk,j(s)=∏j=1k∑l=0Rj-1(Rj-1l)(-1)Rj-1-lΓ(Rj)γ¯Rjexp(1γ¯)E-l-s(1γ¯). (29)
  By applying inverse Laplace transform (LT) after replacing the exponential integral function with ${\text{E}}_{-l-s}\left(\frac{1}{{\bar{\gamma^{\prime }}}_{j}}\right)={\left(\frac{1}{{\bar{\gamma^{\prime }}}_{j}}\right)}^{-l-s-1}\Gamma \left(1+l+s,\frac{1}{{\bar{\gamma^{\prime }}}_{j}}\right)$, the PDF expression of Eq (29) can be obtained as
  fλk(λk)=Ls-1{Mλk(-s)}=∏j=1k[∑l=0Rj-1(Rj-1l)(-1)Rj-1-lΓ(Rj)γ¯Rjexp(1γ¯)×Ls-1{(1γ¯)s-l-1Γ(1+l-s,1γ¯)}] (30)
  where $L_{s}^{-1}\left\{\cdot \right\}$ denotes the inverse LT with respect to *s*. In Eq (30), the inverse LT term can be evaluated by applying the inverse LT pair given in [26, 5.11.(42)] and the frequency shifting property given in [26, 4.1.(5)]. Then, Eq (30) can be finally re-written as the closed-from expression as
  fλk(λk)=∏j=1k[∑l=0Rj-1(Rj-1l)(-1)Rj-1-lΓ(Rj)γ¯Rj×exp(1γ¯)exp((l+1)λk)exp(-exp(-λk)γ¯)] (31)
  Note that for *j* = *k* case, by replacing *R*_*j*_ with *R*_*k*_ + 1, the final result can be obtained.

Based on the above analysis, we observe that the average *AR* depends on *λ*_*k*_ for the given number of active relays at both all previous (*j*-th for 1 ≤ *j* < *k*) and current (*k*-th) subframe. If we let $x_{j}=\sum_{i}^{R_{j}}\gamma_{j,i}$ (*x*_*j*_ ≥ 0), then *λ*_*k*_ for MoDDF_IPS_ and MoDDF_FS_ can be re-written as

$$\begin{array}{c} \lambda_{k,\text{IPS}}=\log\left(1+\sum_{j=1}^{k}x_{j}+\gamma_{\text{SD}}\right) \end{array}$$ (32)

and

$$\begin{array}{ccc} \lambda_{k,\text{FS}} & = & \sum_{j=1}^{k-1}\log(1+x_{j})+\log\left(1+x_{k}+\gamma_{\text{SD}}\right)\\ & = & \log(\left\{\left(1+x_{k}\right)+\gamma_{\text{SD}}\right\}\prod_{j=1}^{k-1}\left(1+x_{j}\right)), \end{array}$$ (33)

respectively. In Eq (33), the internal terms of the logarithmic function can be re-written as

{(1+xk)+γSD}∏j=1k-1(1+xj)=1+∑j=1kxj+γSD+∑n=2k∑{i1,⋯,in}∈Pn(Ik)∏m=1{i1,⋯,in}nxim+γSD(∑n=1k-1∑{i1,⋯,in}∈Pn(Ik-1)∏m=1{i1,⋯,in}nxim) (34)

where we define index set *I*_*k*_ as *I*_*k*_ = {1, 2, ⋯, *k*} and the subset of *I*_*k*_ with *n* (*n* ≤ *k*) elements is denoted by $P_{n}\left(I_{k}\right)$. From Eqs (32) and (34), we can also observe that MoDDF_FS_ provides the better performance than MoDDF_IPS_ over the same channel conditions where the equality holds if and only if *x*_*j*_ = 0 for all *j* (*j* = 1, 2, ⋯, *k*).

## Overhearing Mechanism (ACK/NACK) based MoDDF relaying Protocol with Improved Spectral Efficiency

Based on the system model of the type II relay shown in Fig 1, each UE relay can overhear the reference signal including ACK/NACK signal periodically sent from D to S [1]. Such overheard signals can be used for estimating each R-D link quality. Then, MoDDF relaying protocol can be refined to exploit this limited feedback information such that only those UE relays with relatively better R-D link quality are allowed to forward their decoded messages after correct decoding similar to the on-off based scheduling (OOBS) scheme proposed in [27, 28]. Here, it is necessary to determine the relative strength of ACK/NACK overheard messages at UE relays by comparing it with some pre-determined system threshold, denoted by *γ*_*T*_, so as to identify whether the R-D link quality is acceptable. Then, only by limiting the forwarding UE relays to a subset of such candidates, this overhearing mechanism will certainly improve the spectral efficiency.

### Conditional Average Achievable Rate

In this case, the average *AR* analysis is similar to the previous section. Based on the quality of the R-D link, only relays with better channel among new active relays will participate. Therefore, the distribution of the R-D link SNR becomes a truncated version of the original PDF. In this case, the average *AR* analysis depends on the number of acceptable relays instead of the number of new active relays. As results, the conditional average *AR* in Eq (1), $AR(\bar{\gamma }|\cdot )$, can be re-formulated as a function of the number of acceptable relays at the *j*-th subframe (*j* = 1, 2, ⋯, *N*), *r*_*j*_, as

$$\begin{array}{ccc} & & AR^{\prime }\left(\bar{\gamma }|R_{1},\cdots ,R_{N}\right)\\ & & \;=\sum_{r_{1}}\cdots \sum_{r_{N}}AR(\bar{\gamma }|r_{1},\cdots ,r_{N})\;p_{r_{1},\cdots ,r_{N}|R_{1},\cdots ,R_{N}}\left(r_{1},\cdots r_{N}\right). \end{array}$$ (35)

In Eq (35), *AR* is the same as Eq (10) but it is the function of the number of acceptable relays given the number of new active relay and the joint PMF has the multiple product form of joint PMF of *r*_*j*_ given *R*_*j*_, especially for i.i.d case, as

$$\begin{array}{c} p_{r_{1},\cdots ,r_{N}|R_{1},\cdots ,R_{N}}\left(r_{1},\cdots r_{N}\right)=\prod_{j=1}^{N}p_{r_{j}|R_{j}}\left(r_{j}\right) \end{array}$$ (36)

where

pri|Ri(x)=(Rix)pix(1-pi)Ri-x (37)

and

$$\begin{array}{c} p_{i}=\mathrm{Pr}\left[\gamma_{R}>\gamma_{T}\right]. \end{array}$$ (38)

Now, we also need to derive *f*_*λ*_*k*__(*λ*_*k*_) for each strategy. Here, we can directly apply the similar approaches used in the previous section except the distribution of the R-D link SNR becomes a truncated version of the original PDF. As results, the closed-form expression of each cases can be obtained as follows:

#### MoDDF_IPS_

1. For *k* = 1:
  Since only DT is executed, *f*_*λ*_1__(*λ*_1_) has the same result as given in Eq (14).
2. For *k* > 1:
  In this case, similarly EC may occur at D. The only difference is that only relays with better channel conditions among new active relays will participate. Therefore, the distribution of the R-D link SNR becomes a truncated version of the original PDF, i.e., it follows a conditional PDF of a truncated (above preselected threshold, *γ*_*T*_) RV. In addition, this distribution depends on the number of acceptable relays, *r*_*j*_, instead of the number of new active relays, *R*_*j*_. Therefore, with the help of [28, Eq (5)], *f*_*λ*_*k*__(*λ*_*k*_) can be written as
  $$\begin{array}{c} f_{\lambda_{k}}\left(\lambda_{k}\right)=f_{\gamma_{\text{OOBS}}}\left(e^{\lambda_{k}}-1\right)\cdot e^{\lambda_{k}} \end{array}$$ (39)
  where *f*_*γ*_OOBS__(*γ*) follows a truncated version of the original PDF, *f*_*γ*′_*k*__(*x*) as
  $$\begin{array}{c} f_{\gamma_{\text{OOBS}}}\left(\gamma \right)=\left\{ \begin{array}{cc} \frac{f_{{\gamma^{\prime }}_{k}}\left(\gamma \right)}{1-F_{{\gamma^{\prime }}_{k}}\left(\gamma_{T}\right)} & \text{for}\;\gamma \geq \gamma_{T}\\ 0 & \text{otherwise} \end{array}\right.. \end{array}$$ (40)
  Since *f*_*γ*′_*k*__(*γ*) and *F*_*γ*′_*k*__(*γ*_*T*_) depend on not the number of new active relay but the number of acceptable relays, they follow a Gamma distribution with parameters $\left({N^{\prime }}_{k},\frac{1}{\bar{\gamma }}\right)$ where ${N^{\prime }}_{k}=\sum_{j=1}^{k}r_{j}+1$ as
  $$\begin{array}{c} f_{{\gamma^{\prime }}_{k}}\left(\gamma \right)=\frac{1}{\Gamma \left({N^{\prime }}_{k}\right){\bar{\gamma }}^{{N^{\prime }}_{k}}}\gamma^{{N^{\prime }}_{k}-1}\exp\left(-\frac{\gamma }{\bar{\gamma }}\right) \end{array}$$ (41)
  and
  $$\begin{array}{c} F_{{\gamma^{\prime }}_{k}}\left(\gamma_{T}\right)=\int_{0}^{\gamma_{T}}f_{{\gamma^{\prime }}_{k}}\left(\gamma \right)d\gamma =1-\frac{\Gamma ({N^{\prime }}_{k},\frac{\gamma_{T}}{\bar{\gamma }})}{\Gamma ({N^{\prime }}_{k})}. \end{array}$$ (42)
  As results, for *γ* ≥ *γ*_*T*_, *f*_*γ*_OOBS__(*γ*) and *f*_*λ*_*k*__(*λ*_*k*_) can be obtained as the closed-form expressions as
  $$\begin{array}{c} f_{\gamma_{OOBS}}\left(\gamma \right)=\frac{1}{\Gamma \left({N^{\prime }}_{k},\frac{\gamma_{T}}{\bar{\gamma }}\right){\bar{\gamma }}^{{N^{\prime }}_{k}}}\gamma^{{N^{\prime }}_{k}-1}\exp\left(-\frac{\gamma }{\bar{\gamma }}\right) \end{array}$$ (43)
  and
  $$\begin{array}{ccc} f_{\lambda_{k}}\left(\lambda_{k}\right) & = & \frac{1}{\Gamma \left({N^{\prime }}_{k},\frac{\gamma_{T}}{\bar{\gamma }}\right){\bar{\gamma }}^{{N^{\prime }}_{k}}}{\left(\exp(\lambda_{k})-1\right)}^{{N^{\prime }}_{k}-1}\\ & & \times \exp\left(-\frac{\exp(\lambda_{k})-1}{\bar{\gamma }}\right)\exp\left(\lambda_{k}\right), \end{array}$$ (44)
  respectively.

#### MoDDF_FS_

1. For *k* = 1:
  In this case, *λ*_1_ and *f*_*λ*_1__(*λ*_1_) have the same results as MoDDF_IPS_.
2. For *k* > 1:
  Similarly, *f*_*γ*_OOBS__(*γ*) and *f*_*λ*_*k*,*j*__(*λ*_*k*,*j*_) can be obtained as
  $$\begin{array}{c} f_{\gamma_{\text{OOBS}}}\left(\gamma \right)=\left\{ \begin{array}{cc} \frac{1}{\Gamma \left(R_{j},\frac{\gamma_{T}}{\bar{\gamma }}\right){\bar{\gamma }}^{r_{j}}}\gamma^{r_{j}-1}\exp\left(-\frac{\gamma }{\bar{\gamma }}\right) & \text{for}\;\gamma \geq \gamma_{T}\\ 0 & \text{otherwise} \end{array}\right. \end{array}$$ (45)
  and
  $$\begin{array}{c} f_{\lambda_{k,j}}\left(\lambda_{k,j}\right)=\left\{ \begin{array}{cc} \frac{1}{\Gamma \left(r_{j},\frac{\gamma_{T}}{\bar{\gamma }}\right){\bar{\gamma }}^{r_{j}}}{\left(\exp(\lambda_{k,j})-1\right)}^{r_{j}-1}\\ \times \exp\left(-\frac{\exp(\lambda_{k,j})-1}{\bar{\gamma }}\right)\exp\left(\lambda_{k,j}\right) & \text{for}\;\gamma \geq \gamma_{T}\\ 0 & \text{otherwise} \end{array}\right.. \end{array}$$ (46)
  Then, after applying the binomial expansion, the MGF expression of Eq (46) for *γ* > *γ*_*T*_ can be obtained as
  Mλk,j(s)=∫0∞fλk,j(x)exp(sx)dx=∑l=0rj-1(rj-1l)(-1)rj-1-lΓ(rj,γTγ¯)γ¯rjexp(1γ¯)E-l-s(1γ¯) (47)
  which leads to the final desired form of *f*_*λ*_*k*__(*λ*_*k*_) as
  fλk(λk)=∏j=1k[∑l=0N′k-1(rj-1l)(-1)rj-1-lΓ(rj,γTγ¯)γ¯rj×exp(1γ¯)exp((l+1)λk)exp(-exp(-λk)γ¯)]. (48)

## Results

In this section, we show the performance of two proposed strategies based on MoDDF in terms of *AR* and the average number of channel usage over i.i.d. Rayleigh fading conditions along with the results obtained by Monte-Carlo simulation.

**Fig 5:**
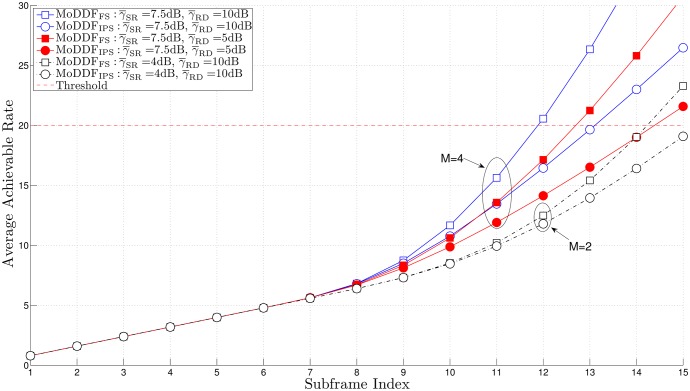
Average achievable rate over a frame when *N* = 15, ${\bar{\gamma }}_{\text{SD}}=2$ dB, and *R*_th_ = 20.

**Fig 6:**
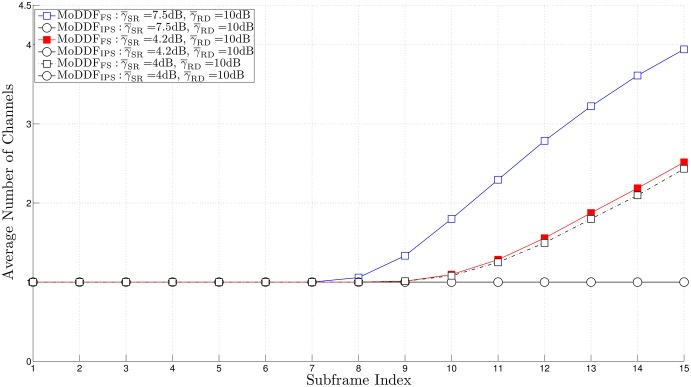
Average channel usage over a frame when *M* = 7, *N* = 15, ${\bar{\gamma }}_{\text{SD}}=2$ dB, and *R*_th_ = 20.

From Fig 5, similar to the channel capacity comparison result in [20], we can observe that MoDDF_FS_ provides the better rate than MoDDF_IPS_ and this performance gap increases as the quality of both S-R and R-D links increase. For example, as the quality of S-R link increases, the possibility of more relays participating in the retransmission is increasing. Note that based on the proposed strategies, for MoDDF_FS_, DT plus all types of combining (EC, IC, and MC) are randomly performed at D while for MoDDF_IPS_, DT plus only one type of combining (EC). Here, as this possibility increases, for MoDDF_FS_, the possibility of IC or MC being performed increases while for MoDDF_IPS_, only EC is performed. As a result, from Theorem 3.1 in [20], we can claim that MoDDF_FS_ provides the better data rate performance than MoDDF_IPS_ at the cost of using more channel resources. In MoDDF, the performance ranges between MoDDF_FS_ and MoDDF_IPS_ depending on channel conditions. More specifically, the performance depends on how the signals are combined, i.e., EC, IC, and MC. For an incremental rate, as the quality of the S-R link increases, the performance of MoDDF_FS_ increases faster than that of MoDDF_IPS_. In terms of the channel usage in Fig 6, MoDDF_IPS_ always uses only single channel while MoDDF_FS_ uses multiple channels. However, in MoDDF_IPS_ under interference-limited condition, a serious interference problem occurs because the retransmission from all active Rs is performed through the single channel. In practice, sharing a single channel among multiple relays may cause the performance degradation.

**Fig 7:**
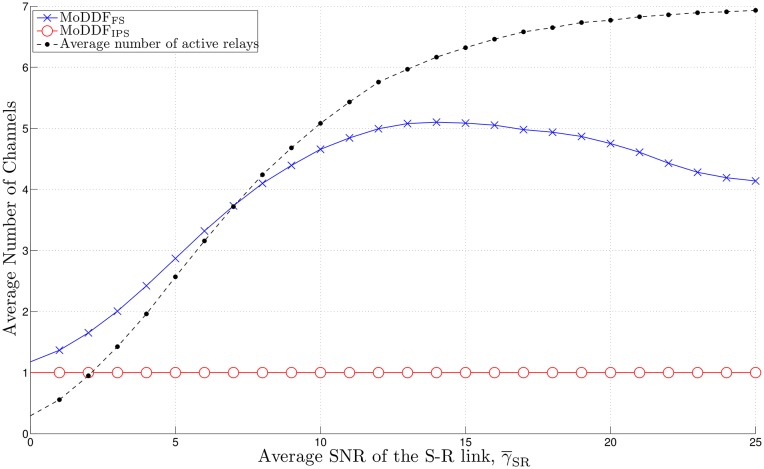
Maximum channel usage per single subframe when *M* = 7, *N* = 15, and *R*_th_ = 20.

Fig 7 shows the maximum channel usage per single subframe as a function of the average SNR $\bar{\gamma }$ of the S-R link, ${\bar{\gamma }}_{\text{SR}}$. As ${\bar{\gamma }}_{\text{SR}}$ increases, the maximum number of channels being occupied by active relays in MoDDF_FS_ increases up to a certain point which depends on channel conditions. However, as the number of active relays is continuously increasing beyond that point, the possibility of having as many relays being activated simultaneously at a subframe is also increased, and based on the proposed strategies of MoDDF_FS_, these relays tend to share the same channel at a subframe, which in turn reduces the possibility of the maximum number of channels being used simultaneously.

**Fig 8:**
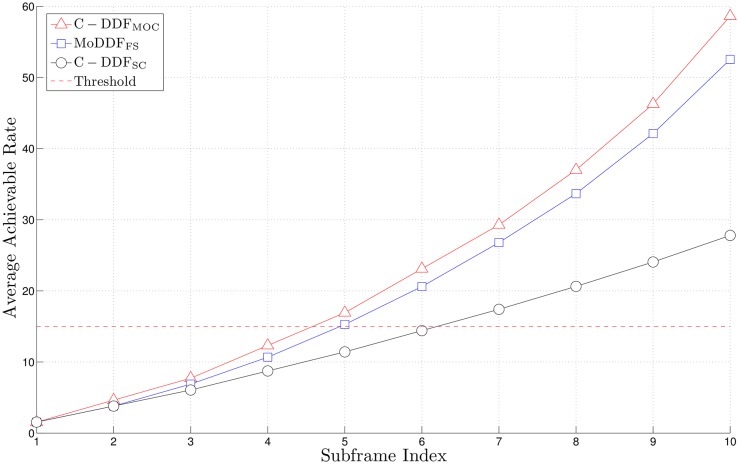
Average achievable rate over a frame with *M* = 7, *N* = 10, and $\bar{\gamma }=7.4$ dB.

Fig 8 shows the performance comparison between MoDDF and conventional DDF (C-DDF) in terms of the average *AR* over a frame. We only consider MoDDF_FS_ which provides the better performance while MoDDF_IPS_ is the simplest method but provides the lower rate. In order for fair performance comparison between MoDDF_FS_ and C-DDF, two cases for C-DDF are considered as follows: i) C-DDF with same frame structure and single channel/code (SC), C-DDF_SC_ and ii) C-DDF with same frame structure but multiple orthogonal channels/codes (MOC), C-DDF_MOC_. In term of a successful decoding at D, for MoDDF_FS_, D succeeds in decoding after receiving 5-th subframe while for C-DDF_SC_, D succeeds in decoding after receiving 7-th subframe. As a result, the proposed MoDDF can reduce 2 redundant subframes over a frame, which saves the energy by about 8% compared to C-DDF_SC_. For both C-DDF_MOC_ and MoDDF, the early termination of decoding can be activated similarly. However, MoDDF uses relatively fewer channels while C-DDF_MOC_ always requires a full channel usage [20]. Moreover, C-DDF_MOC_ may not be suitable for our target system (e.g., type II relays) due to the need of exchanging control messages between relays and the end user which violates the transparency requirements.

In Figs 5, 6 and 8, we can observe that the early termination at the end user can be made available at D, so that a certain number of subframes over a frame can be saved and eventually, a considerable number of subframes can be saved in full-rate data transmission.

**Fig 9:**
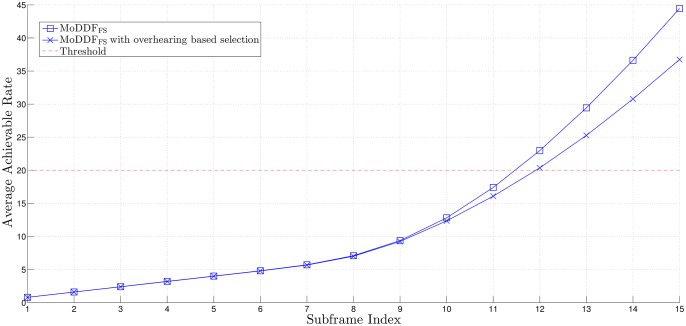
Average achievable rate over a frame when *M* = 7, *N* = 15, ${\bar{\gamma }}_{\text{SD}}=2$ dB, *R*_th_ = 20, and *γ*_*T*_ = 20 dB.

**Fig 10:**
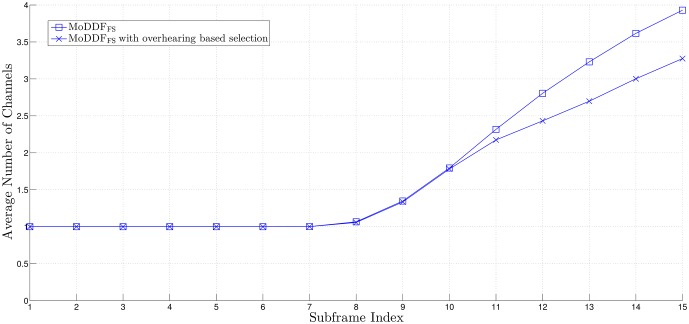
Average channel usage over a frame when *M* = 7, *N* = 15, ${\bar{\gamma }}_{\text{SD}}=2$ dB, *R*_th_ = 20, and *γ*_*T*_ = 20 dB.

Further, in Figs 9 and 10 we observe that the required data rate can be achieved with the same number of subframes but less channel resources with the help of the overhearing mechanism although only the acceptable relays among the new active relays participate in the retransmission. As a result, limiting the forwarding UE relays based on the overhearing mechanism can certainly lead the improvement of the spectral efficiency while still providing satisfactory performance.

## Conclusions

In this paper, we have proposed MoDDF which can be applied to the type II relay for the next-generation cellular systems (e.g., LTE-Advanced and beyond). In particular, the end user can perform the joint and sequential decoding without exchanging the control message with UE relays, because it does not need to know whether a UE relay is participating in the transmission (i.e., appears transparent to the end user) or not. In addition, the performance of the data rate at the end user in an indoor wireless environment or near cell boundary can be improved as an additional benefit through the random combination of EC, IC, and MC across subframes that are offered by the joint and sequential decoding implemented in this paper. Further, enabling the early termination of decoding at the end user can provide a considerable resource saving, resulting in the higher spectral and energy efficiency. Besides, MoDDF_FS_ can be applied to interference-limited environment, assuring reliable performance via the early termination.

In this work, for the analytical tractability, we have assumed that all channels (both S-D and S-R-D) experience quasi-static i.i.d. Rayleigh fadings. However, in practice, every path may not be i.i.d.. As one of common possible scenarios, we can consider a non-identical case. More specifically, the possibility of successfully decoding at R is increasing as the SNR of the S-R link increases while the possibility of successful decoding at D is increasing as the SNR of the R-D link increases which may directly affect the result of the data rate improvement at D. Note that, even if our results are based on the identical assumption, our results can still be used as an upper bound on the performance.

The proposed scheme in this paper can be applied to one of potential solutions for UE relay based public safety where the end user is out of coverage [17] by enabling UE-to-UE direct communication with proximity. More specifically, by iteratively scheduling a relatively better UE relay closer to the end user in a probabilistic sense, one in each subframe, a temporary communication network can be established with a route consisting of a sequence of these candidate relays. As a result, it may be possible to provide the reliable communication over open-loop access link which may be possible from the disaster region or any nearby regions.
